# Chronic Subordinate Colony Housing (CSC) as a Model of Chronic Psychosocial Stress in Male Rats

**DOI:** 10.1371/journal.pone.0052371

**Published:** 2012-12-26

**Authors:** Kewir D. Nyuyki, Daniela I. Beiderbeck, Michael Lukas, Inga D. Neumann, Stefan O. Reber

**Affiliations:** Department of Behavioural and Molecular Neurobiology, University of Regensburg, Regensburg, Germany; University of California, Los Angeles, United States of America

## Abstract

Chronic subordinate colony housing (CSC) is an adequate and reliable mouse model of chronic psychosocial stress, resulting in reduced body weight gain, reduced thymus and increased adrenal weight, long-lasting anxiety-like behaviour, and spontaneous colitis. Furthermore, CSC mice show increased corticotrophin (ACTH) responsiveness to acute heterotypic stressors, suggesting a general mechanism which allows a chronically-stressed organism to adequately respond to a novel threat. Therefore, the aim of the present study was to extend the CSC model to another rodent species, namely male Wistar rats, and to characterize relevant physiological, immunological, and behavioural consequences; placing particular emphasis on changes in hypothalamo-pituitary-adrenal (HPA) axis responsiveness to an acute heterotypic stressor. In line with previous mouse data, exposure of Wistar rats to 19 days of CSC resulted in a decrease in body weight gain and absolute thymus mass, mild colonic barrier defects and intestinal immune activation. Moreover, no changes in stress-coping behaviour or social preference were seen; again in agreement with the mouse paradigm. Most importantly, CSC rats showed an increased plasma corticosterone response to an acute heterotypic stressor (open arm, 5 min) despite displaying similar basal levels and similar basal and stressor-induced plasma ACTH levels. In contrast to CSC mice, anxiety-related behaviour and absolute, as well as relative adrenal weights remained unchanged in CSC rats. In summary, the CSC paradigm could be established as an adequate model of chronic psychosocial stress in male rats. Our data further support the initial hypothesis that adrenal hyper-responsiveness to ACTH during acute heterotypic stressors represents a general adaptation, which enables a chronically-stressed organism to adequately respond to novel challenges.

## Introduction

In humans, chronic stress has been repeatedly shown to be a risk factor for the development of several affective and somatic disorders (for review see [1], [2]). There is also a large body of evidence from rodent studies indicating a link between chronic or repeated stress and emotional, social and physiological, in particular immunological, dysfunctions [3]–[7]. However, despite this knowledge and substantial research efforts in the last decades, the aetiology of stress-based disorders remains poorly understood. This has led to a resurgence of interest in developing more clinically relevant animal models of chronic stress. Given the increasing evidence for chronic psychosocial stress being a risk factor for the development of stress-related pathologies in humans (for review see [8], [9]), recent attempts have focused on the development of novel psychosocial stress paradigms believed to better mimic the human situation [4], [5], [10], [11].

We have recently established chronic subordinate colony housing (CSC) as a clinically relevant mouse paradigm for chronic psychosocial stress [12], [13]. During CSC exposure, 4 experimental male mice are housed together with a dominant, and slightly larger resident for 19 consecutive days, whereby, the larger male is replaced by a novel one on days 8 and 15 to avoid habituation [12]. Reliable indicators of chronic stress in CSC mice are a decrease in body weight gain and thymus weight, an increase in adrenal mass, development of spontaneous colitis and aggravation of a chemically-induced colitis, increased anxiety-related behaviour, but no changes in sucrose consumption/preference and immobility in the forced swim and tail suspension test [12]–[15]. Interestingly, one key factor involved in the development of CSC-induced spontaneous colitis has been shown to be bacterial translocation, endorsed by a leaky colonic barrier caused at least partly by a decrease in colonic mucus production [16]. CSC mice further show an increased risk for inflammation-related colon carcinogenesis [17]. Finally, CSC affects adrenal functionality resulting in unaffected basal morning, but decreased basal evening, plasma corticosterone levels [12], and a reduced adrenal *in vitro* ACTH responsiveness [12], [18]. These findings, at least at the first glance, suggest the development of adrenal insufficiency and, thus, seem to be in line with the negative immunological and behavioural consequences of CSC in mice.

However, in contrast to the reduced adrenal *in vitro* ACTH responsiveness, we recently showed an increased *in vivo* plasma corticosterone response to an acute heterotypic stressor, namely exposure to an elevated platform, in CSC compared with single-housed control (SHC) mice [18]. As a comparable rise in plasma ACTH was found, CSC-induced changes at the level of the adrenal gland are likely and may include decreased *in vitro* ACTH responsiveness, but increased *in vivo* ACTH sensitivity during acute heterotypic stressors. Such changes might represent beneficial adaptations to, rather than maladaptive consequences of, chronic psychosocial stress, allowing an adequate response to a novel challenge while preventing prolonged exposure to high basal levels of deleterious corticosterone. Attenuated responses of the HPA axis to repeated homotypic (for review see [19]), but sensitization to acute heterotypic stressors has been repeatedly described in mice and rats [20]–[23]. However, in contrast to our findings in CSC mice, until today, there is the general assumption that these adaptations are not relevant for stressors which are of social nature [24]. Therefore, the biological relevance of our mouse data needs to be further substantiated and confirmed in a different rodent species.

In the present study, we therefore established the CSC model in male Wistar rats grouped into CSC, single-housed (SHC), and group-housed (GHC) controls. In order to confirm that the male Long-Evans rats used as residents obtained the dominant, and the 4 Wistar rats the subordinate positions during CSC exposure, we monitored their offensive and defensive behaviours. Next, we investigated the effects of CSC on well established stress parameters such as body, thymus and adrenal weight, and basal plasma corticosterone levels. Using repeated jugular vein sampling via chronically implanted catheters we further monitored plasma ACTH and corticosterone responses to an acute emotional stressor (open arm exposure). We also assessed CSC effects on socio-emotional behaviours (anxiety-related and stress-coping behaviours, social preference, inter-male aggression) and on somatic symptoms (signs of colonic barrier defects and of colonic inflammation).

## Materials and Methods

### Ethics Statement

All experimental protocols were approved by the Committee on Animal Health and Care of the local government of Oberpfalz (54–2531.2–16/08), and conformed to international guidelines on the ethical use of animals. Surgery was performed under isoflurane anesthesia, and all efforts were made to minimize the number of animals used and their suffering.

### Animals

Male Wistar (300–350 g; experimental rats), and male (400–450 g; dominant rats) and female (300–350 g) Long-Evans rats were purchased from a commercial supplier (Charles River, Sulzfeld, Germany) and kept under standard laboratory conditions (12/12-h light–dark cycle, lights on at 0600 h, 21±1°C, 60±5% humidity, free access to tap water and standard rat chow). Each Long-Evans resident male was permanently housed with a Long-Evans female in order to stimulate territorial behaviour and improve offensive approach [25], and single-housed 1 h before the start of CSC colony formation (see below). All experimental Wistar rats were randomly housed in groups of 4 upon arrival for 1 week to allow habituation.

### Chronic Subordinate Colony Housing (CSC)

**Figure 1:**
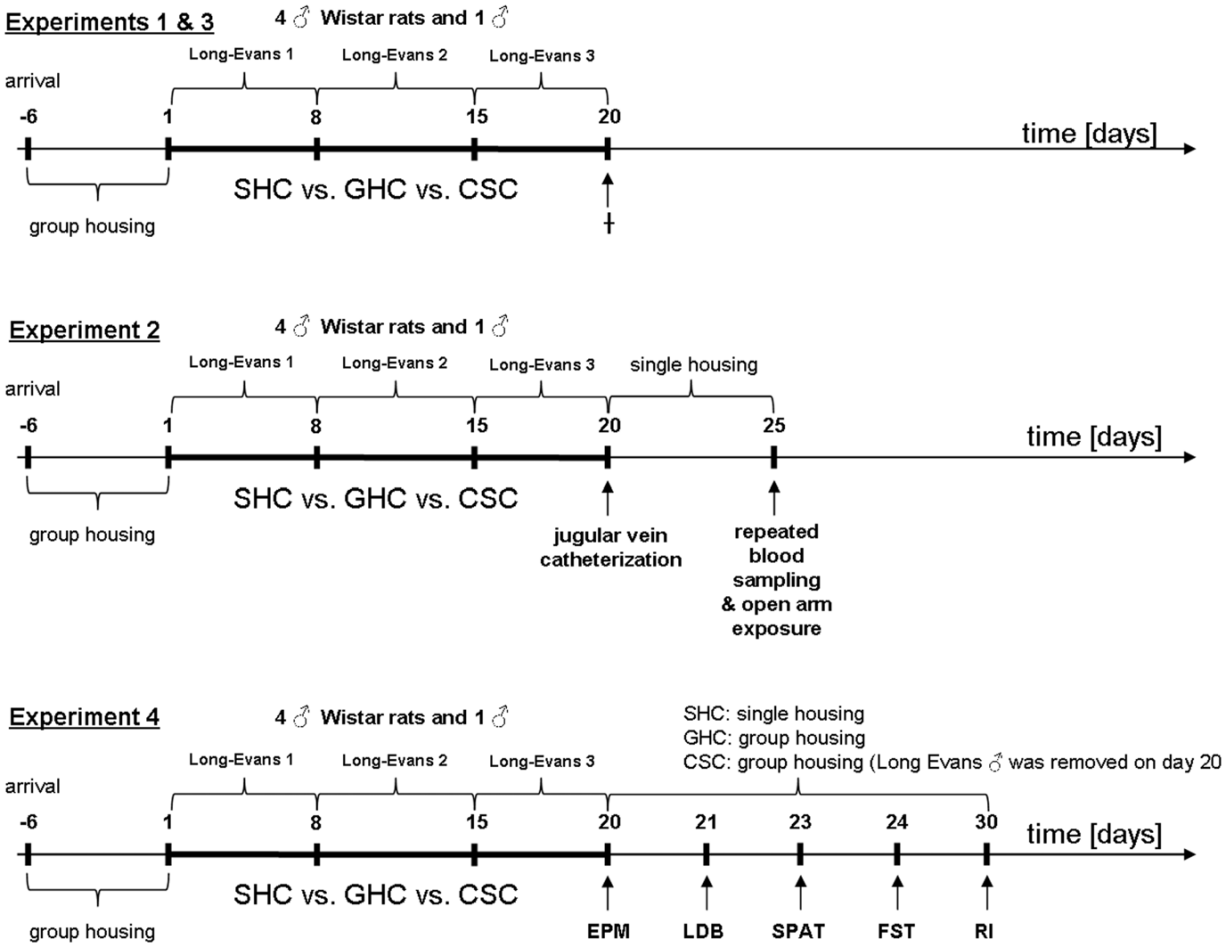
Experimental design. Male Wistar rats were exposed to 19 days of chronic psychosocial stress, i.e. chronic subordinate colony housing (CSC). Thereby, 4 male CSC rats were housed with a dominant Long-Evans male, which was replaced by a novel dominant male on days 8 and 15 to avoid habituation. Single-housed and group-housed (4 per cage) male rats served as controls i.e., SHC and GHC respectively. In experiments 1 and 3, all rats were killed (†) in the morning of day 20, in order to investigate the effects of CSC exposure on relevant physiological and immunological parameters. In experiment 2, catheters were implanted in the right jugular vein of all rats in the morning of day 20, after which they were single-housed in novel plexiglas observation cages. Five days after catheterization (day 25), rats were exposed to an acute emotional stressor (open arm; 5 min) in order to investigate plasma ACTH and corticosterone responsiveness. In experiment 4, dominant Long-Evans rats were removed from CSC colonies after assessing anxiety-related behaviour on the elevated plus-maze (EPM) on day 20. Anxiety-related behaviour was again assessed in the light-dark box (LDB) the next day (day 21). On days 23, 24 and 30, social preference/avoidance, stress-coping behaviour, and inter-male aggression were analyzed using the social preference/avoidance test (SPAT), forced swim test (FST) and resident intruder (RI) test, respectively.

The chronic subordinate colony housing (CSC) paradigm in rats was adapted from the CSC paradigm previously described in mice [12]. Briefly, 4 male Wistar rats were housed together with a larger male Long-Evans rat in its home cage (56×39×20 cm) for 19 consecutive days (day 1 to day 20). Prior to CSC, the male Long-Evans rats were tested for their aggressive behaviour. During 2 training sessions, the female Long-Evans rats were separated from their male counterparts 1 h before lights off. In the dark phase, 4 unfamiliar male Wistar rat intruders were then introduced to each resident Long-Evans rat for about an hour. The offensive behaviours of residents were observed. Non dominant (non offensive) as well as males that started to injure and bite their opponents were excluded. To avoid habituation, the 4 CSC rats were introduced into the home cage of a novel dominant Long-Evans male on days 8 and 15. During the first 2 h of colony formation which was started immediately after lights off on days 1, 8, and 15, the colonies were videotaped for behavioural analysis. As the appropriate and stress free housing condition for male control rats is still under debate, both SHC and GHC rats were used for comparison. SHC rats were housed singly (40×25×15 cm), whereas GHC rats were housed in groups of four per cage (55×35×20 cm) (Figure 1). All rats were reassigned on day 1 to one of the three housing conditions (e.g. novel GHC groups were formed) so that the mean body weight was comparable. Both control groups were kept in separate animal rooms from the CSC colonies and remained undisturbed in their home cages except for change of bedding once a week. For assessment of body weight, all rats were weighed in the morning of days 1, 3, 8, 10, 15, 17 and 20, and delta body weight gain between day 20 and day 1 was calculated.

### Experimental Procedures

#### Experiment 1

In order to confirm the consequences of CSC exposure on established physiological and HPA axis parameters in male rats, SHC, GHC and CSC rats were rapidly killed by decapitation (after brief inhalation anesthesia) in the morning of day 20 of CSC exposure between 0800 h and 1000 h (Figure 1). Trunk blood was collected for the quantification of plasma corticosterone levels. Moreover, changes in adrenal and thymus weight were determined (see below). The behaviour during the first 2 h after CSC colony formation was recorded for later analysis of offensive and defensive behavioural patterns.

#### Experiment 2

In order to investigate plasma ACTH and corticosterone responses to an acute emotional stressor, another set of SHC, GHC and CSC rats, fitted with a chronic jugular vein catheter on day 20 were immediately single-housed in novel cages thereafter. Five days later (day 25), they were exposed to the open arm of an elevated plus-maze for 5 min. Blood was repeatedly drawn under basal conditions and 5, 15 and 60 min after termination of open arm exposure (Figure 1).

#### Experiment 3

In order to confirm that CSC exposure also results in colonic inflammation in rats as shown in mice [12], [16], another set of SHC, GHC and CSC rats was killed in the morning of day 20 (Figure 1). Colon and mesenteric lymph nodes were removed to determine the histological damage score, colonic mucus production (Alcian Blue staining) and anti-CD3-stimulated interferon-gamma (IFN-γ) secretion from mesenteric lymph node cells (see below).

#### Experiment 4

To study the behavioural consequences of CSC in rats, another set of SHC, GHC and CSC rats was subjected to a series of behavioural tests on the last day of CSC (elevated plus-maze) and following termination of CSC, respectively. Whereas the housing conditions for SHC and GHC remained unchanged until termination of all behavioural tests (until day 30), the dominant residents were removed from the CSC colonies on day 20 after elevated plus-maze testing, and CSC rats remained in groups of 4 until termination of all behavioural tests (until day 30). The effects of CSC on anxiety-related behaviour were investigated on the elevated plus-maze and in the light-dark box on days 20 and 21, respectively. In addition, CSC effects on social anxiety were assessed employing the social preference/social avoidance test on day 23, i.e. 3 days after termination of CSC. Furthermore, stress-coping behaviour and inter-male aggression were investigated in the forced swim test and the resident-intruder test on days 24 and 30, respectively (Figure 1).

### Behavioural Observations during CSC

To confirm the intended dominant/subordinate hierarchy within each colony, rats of Experiment 1 were videotaped during the first 2 h of colony formation immediately after lights off on days 1, 8, and 15. Their agonistic behaviour was analyzed in terms of number of offensive behavioural patterns like threat, attack, offensive upright and keep down, and number of defensive behavioural patterns like freezing, defensive upright and lying on the back. Importantly, in each colony, resident Long-Evans rats only showed offensive behavioural elements, and Wistar CSC rats only showed defensive behavioural elements. The mean number of occurrence of each behaviour during the 3×2-h (6-h) observation period was calculated and depicted in the graphs.

### Blood Sampling and Enzyme-Linked Immunosorbent Assay for Corticotrophin and Corticosterone

To determine the effect of CSC on plasma ACTH and corticosterone concentrations, SHC, GHC and CSC rats were either rapidly killed by decapitation under carbon dioxide anaesthesia within 3 min after entering the animal room (Experiment 1; basal plasma corticosterone), or chronically implanted with a jugular vein catheter for repeated blood sampling (Experiment 2; ACTH and corticosterone response to acute stressor). About 100 µl of trunk blood (Experiment 1) or 200 µl of venous blood obtained via the implanted jugular vein catheter (Experiment 2) was collected in EDTA-coated tubes on ice (Sarstedt Nümbrecht, Germany) and centrifuged at 4°C (5000 rpm, 10 min). Plasma samples were stored at –20°C until assayed using commercially available Enzyme-linked Immunosorbent Assay kits for ACTH (analytical sensitivity 0.22 pg/ml, intra-assay and inter-assay coefficients of variation ≤7.1%, IBL International, Hamburg, Germany) and corticosterone (analytical sensitivity <1.631 nmol/l, intra-assay and inter-assay coefficients of variation ≤6.35%, IBL International, Hamburg, Germany). Enzyme-linked Immunosorbent Assay kits with different LOT numbers were used for experiments 1 and 2.

### Determination of Thymus and Adrenal Weights

To assess CSC effects on thymus and adrenal weight, thymus and both adrenal glands were removed after decapitation, carefully pruned from fat tissue and weighed (Experiment 1). The left and right adrenals were weighed together. All values represent absolute (mg) or relative (mg organ weight/g body weight) weights.

### Jugular Vein Catheterization and Repeated Blood Sampling

Implantation of the jugular vein catheter in Experiment 2 was performed as described previously [26], [27] on day 20. Briefly, the rat was placed in a perspex box to allow inhalation of isoflurane anaesthesia, and then transferred to the surgery table, where anaesthesia was maintained via a face mask. The right jugular vein was exposed by blunt dissection, and a catheter consisting of a silicone tubing (Dow Corning Corp., Midland MI, USA) and a polyethylene-50 tubing was inserted approximately 3 cm into the vessel through an incision in a cardiac direction and exteriorized at the neck of the animal behind the ears. The catheter was filled with sterile saline containing gentamicin (30,000 IU/ml; Centravet, Bad Bentheim, Germany). Catheterized animals were then single-housed in standard polycarbonate observation cages (38×22×36 cm) for 5 days (Experiment 2).

In the morning of day 25 at 0700 h, i.e. 5 days after termination of CSC exposure, the catheter of each rat was attached to an extension tube connected to a 1-ml plastic syringe filled with sterile heparinized 0.9% saline (30 IU/ml, Heparin-Natrium, Ratiopharm, Ulm, Germany). Each rat was then left undisturbed for 90 min. Two basal blood samples were taken 30 min apart, before rats were placed on the open arm for 5 min. Subsequent samples were collected 5, 15 and 60 min after end of open arm exposure. Collected blood was immediately replaced with sterile 0.9% saline. All blood samples were collected in EDTA-coated tubes on ice and treated as explained above.

The two basal ACTH and corticosterone concentrations (basal 1 and 2) were averaged to calculate the mean basal concentrations for both ACTH and corticosterone which was set to 100%. Data are presented as percentage increase with respect to basal values.

### Determination of the Histological Damage Score of the Colon

Assessment of the histological damage score was performed as previously described [12], [13]. In order to assess the effect of CSC on histological markers of the distal part of the intestinal tract, the colon was removed and mechanically cleaned (Experiment 3). Afterwards, 1 cm of the distal third of the colon was cut longitudinally, laid on a filter paper and fixed in 10% formalin overnight. The next day, the fixed tissue was embedded in paraffin and cut longitudinally. Two 3 µm haematoxylin-eosin stained sections taken at 100 µm distance were evaluated by histological scoring performed by an investigator blind to treatment. For statistics, each individual score represented the mean of the 2 sections. Histological damage score ranges from 0 to 8 and represents the sum of the epithelium score (0: normal morphology; 1: loss of goblet cells; 2: loss of goblet cells in large areas; 3: loss of crypts; 4: loss of crypts in large areas) and infiltration score (0: no infiltration; 1: infiltrate around crypt bases; 2: infiltrate reaching to lamina muscularis mucosae; 3: extensive infiltration reaching the lamina muscularis mucosae and thickening of the mucosa with abundant oedema; 4: infiltration of the lamina submucosa).

### Alcian Blue Staining

One aspect of epithelial barrier function, namely the number of mucus producing epithelial cells, was investigated as previously described [16]. Briefly, formalin-fixed colon tissue was embedded in paraffin and cut longitudinally (Experiment 3). Two 3-µm sections of each rat were taken at 100-µm distance and used for acidic Alcian Blue staining. Afterwards Alcian Blue positive cells were counted in the colonic crypt layer of ten non overlapping adjacent fields of view (Leika FW4000 software; magnification 1∶20) per section (total of 20 fields of view per rat) and averaged per rat.

### Isolation and Incubation of Mesenteric Lymph Node Cells

Mesenteric lymph nodes from 4 rats per treatment group (randomly selected) were harvested under sterile conditions and collected on ice in cell culture medium [RPMI-1640 supplemented with 10% fetal calf serum (Biochrom, Germany), 100 U/ml penicillin and 100 µg/ml streptomycin (GIBCO-BRL, Eggenstein, Germany) and 3×10^−5^ M ß-mercaptoethanol (Sigma, Deisenhofen, Germany)] for each individual rat (Experiment 3). Lymph nodes were mechanically disrupted and filtered through a cell strainer (70-µm Nylon, Falcon™, Becton Dickinson, Germany). Afterwards, cells were washed three times in cell culture medium and adjusted to a concentration of 10^6^ cells/ml. 2×10^5^ (200 µl) lymph node cells were transferred to wells of a 96-well plate and stimulated by pre-coating wells with 100 µl of 2.5 µg/ml anti-CD3 antibody (final concentration 100 U/ml). Four wells were transferred with the respective number of cells of each rat. After incubation for 24 h (37°C, 5% CO_2_), IFN-γ concentrations were measured in the supernatants of each of the four wells per rat using the MILLIPLEX® MAP Kit for Rat cytokine/chemokine assay (Millipore GmbH, Schwalbach, Germany) and averaged per rat.

### Elevated Plus-maze

To assess the effects of CSC on anxiety-related behaviour, SHC, GHC and CSC rats of Experiment 4 were transported to the elevated plus-maze room the evening before testing, i.e. on day 19. The next day, they were tested on the elevated plus-maze between 0800 and 1200 h for 5 min as described before [28], [29]. The elevated plus-maze consisted of two opposing open (50×10 cm; 100 lux) and closed (50×10×40 cm; 20 lux) arms connected by a central platform (10×10 cm) and elevated 70 cm above the floor. Each rat was placed on the central platform facing a closed arm and allowed to explore the maze for 5 min. Before each test, the maze was thoroughly cleaned. The time spent in the respective arms was recorded by means of a video/computer setup to allow calculation of the percentage of time spent on the open arms of the maze. Further, the number of entries into the closed arms was recorded as a measure of locomotor activity.

### Light-Dark Box

To further assess the effects of CSC on anxiety-related behaviour in another established test [30], SHC, GHC and CSC rats were tested in the light-dark box (Experiment 4) in the morning of day 21 between 0800 and 1200 h as described before [29], [31]. Briefly, the light-dark box consisted of a brightly lit (40×50 cm; 350 lux) and a dark (40×30 cm; 70 lux) compartment separated by a partition wall that had an opening (7.5×7.5 cm) at floor level. Rats were individually placed in the dark box facing the opening and allowed free exploration for 5 min. The time spent in the respective compartments was recorded by means of a video/computer setup to allow calculation of the percentage of time spent in the lit box. The number of line crossings/minute in the dark compartment was taken as an indicator of locomotor activity. The light–dark box was cleaned thoroughly before each test.

### Social Preference/avoidance Test

The effects of CSC on social anxiety were studied using the social preference/avoidance test in Experiment 4 as described before in rats and mice [15], [32]. Briefly, 1 h after lights off (1900 h) on day 23, SHC, GHC and CSC rats were habituated in a novel arena (40×80×40 cm, red light) for 30 s, then an empty wire-mesh cage (non-social stimulus) was placed at one side wall of the arena for 4 min. The empty cage was then exchanged by an identical cage containing an unknown male con-specific (social stimulus) for an additional 4 min. Each test procedure was videotaped and scored afterwards by an observer blind to treatment using JWatcher behavioural observation software (V 1.0, Macquarie University and UCLA). Non-social and social stimulus investigation times were scored by measuring the time the rat spent in active olfactory investigation (sniffing). Social preference/avoidance was calculated based on the following formula: (time investigating the social stimulus [s]/time investigating the empty cage [s]) ×100%. A value less than 100% was taken to represent social avoidance, while a value above 100% was taken to indicate social preference. The arena was cleaned thoroughly before each test.

### Forced Swim Test

In order to assess the effects of CSC on stress coping, SHC, GHC and CSC rats were tested in the modified forced swim test as described before [33], which was originally established by Porsolt and colleagues for screening anti-depressants [34]. Briefly, between 0800 and 1100 h of day 24 (Experiment 4), rats were individually placed into a plexiglas tank (50 cm high × 29 cm in diameter) filled with 25°C water to a depth of 30 cm for 10 min. Each session was recorded using a video camera placed in front of the cylinder for subsequent analysis. Afterwards, the behaviours floating, struggling and swimming were manually quantified in a 5-s interval in the 600-s test period (total of 120 scores/events) by an observer blind to treatment. Water was changed before each trial. The animal was removed, towel-dried and placed in a clean observation plexiglas cage (40×24×36 cm) for a 2-h recovery period.

### Resident-Intruder Test

In order to assess aggressive behaviour in SHC, GHC and CSC rats (Experiment 4), we employed the resident intruder test as described before [35], [36] on day 30. Therefore, after the 2-h recovery period on day 24, SHC, GHC and CSC rats (known as residents in this test) in the plexiglas cages were transported to the neighboring experimental room and each housed with a sexually receptive female for 6 days in order to stimulate territorial behaviour [25]. The 12/12-h light-dark cycle switched to lights off at 1200 h. The resident-intruder test was performed on day 30 in the early dark phase (1 h after lights off), which is the most active phase in rats. At about 1300 h, the female was removed 30 min before the onset of the resident-intruder test. An unfamiliar, lighter (∼10%) male con-specific was placed in the resident’s home cage for 10 min. The test was videotaped and afterwards the aggressive behavioural patterns of the residents were scored by an experienced observer blind to treatment as explained in detail before [36]. These patterns consisted of attack, lateral threat, offensive upright, keep down, threat, aggressive grooming and were scored in real-time using preset keys on a computer (Eventlog; Version 1.0, 1986, R. Hendersen) and calculated as percentage of overall test time. “Lateral threat” and “keep down” was defined as described by Koolhaas and colleagues [37], meanwhile ‘’threat’’ was defined in a similar way as “lateral threat” but lacking the lateral approach [38]. “Offensive upright” and “aggressive grooming” was defined as offensive versions of the upright posture and grooming, respectively, again as described by Koolhaas and colleagues [37]. Further, “attack” was defined as hostile approach of the opponent. Additionally, the attack latency and number of attacks were counted.

### Statistical Analysis

For statistical analyses, the software package SPSS 18.0 (SPSS Inc., Chicago, IL, USA) was used. The following tests were used: the Mann-Whitney U test for behavioural analysis during CSC, the two-way analysis of variance (ANOVA) for repeated measures for ACTH and corticosterone levels in blood samples obtained from catheterized rats (factors treatment x time), and the one-way ANOVA (factor treatment) for all other readouts. Both ANOVA were followed by *Bonferroni post hoc* test if appropriate. Data are presented as mean ± SEM; P≤0.05 was considered statistically significant.

## Results

### Experiment 1

#### Behavioural analysis during CSC

**Figure 2:**
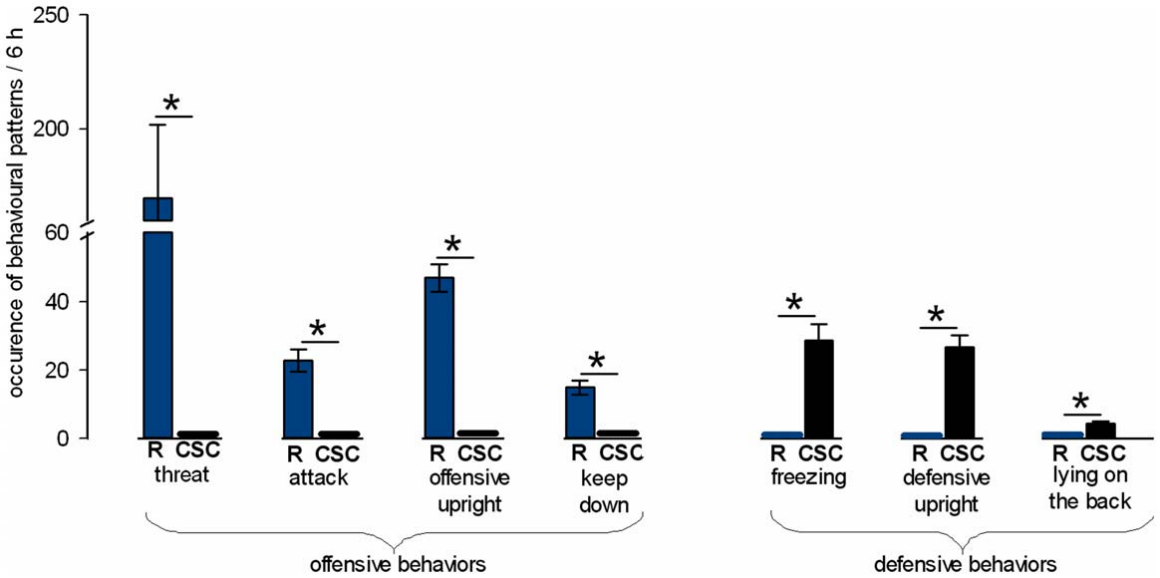
Figure 2. Offensive and defensive behavioural patterns displayed during chronic subordinate colony housing (CSC). Mean offensive and defensive behavioural patterns displayed by the Long-Evans residents (R; blue columns) and the CSC (black columns) rats during the first 2 h of CSC colony formation on days 1, 8 and 15 (3×2 h = 6 h). Long-Evans residents (n = 27) only showed offensive behaviours including threat, attack, offensive and keep down. CSC rats (n = 35) only displayed defensive and submissive behaviours such as freezing, defensive upright and lying on the back. Data represent means ± S.E.M. * P<0.05 vs respective behaviour shown by R.

During the first 2 h of colony formation on days 1, 8 and 15 of CSC exposure, only the larger male Long-Evans rats displayed offensive behaviours (Figure 2), thus establishing the dominant position in each CSC colony. In contrast, CSC male Wistar rats only displayed defensive behaviours resulting in subordinate positions in each colony. Consequently, the numbers of threats, attacks, offensive uprights and keep downs shown by the dominant Long-Evans rats were significantly higher compared with CSC rats (P<0.001). *Vice versa*, CSC rats displayed significantly more freezing, defensive upright, and lying on the back postures compared with Long-Evans residents (P<0.001; Figure 2).

#### CSC effects on body weight gain, absolute thymus and adrenal weight and basal plasma corticosterone

**Figure 3:**
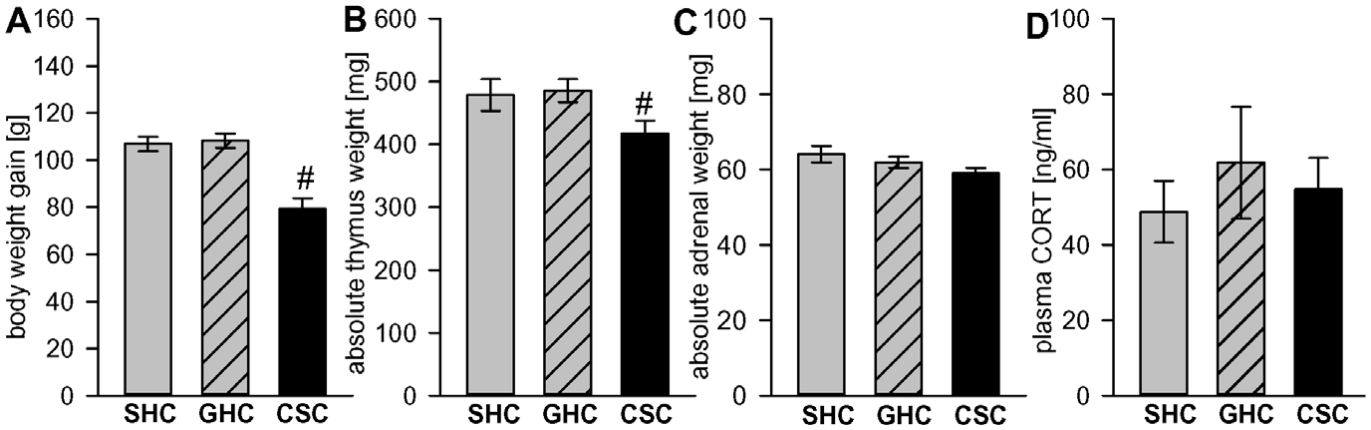
Figure 3. Effects of CSC on body weight gain, absolute thymus and adrenal weight, and basal plasma corticosterone. Nineteen days of CSC exposure (n = 35) resulted in a decreased body weight gain (A) and decreased absolute thymus weight (B) compared with single-housed controls (SHC, n = 25) and group-housed controls (GHC, n = 40), but caused no changes in absolute adrenal weight (C) and basal plasma corticosterone (D). Data represent means ± S.E.M.; # p<0.05 versus SHC and GHC.

Statistical analysis revealed that CSC compared with both SHC and GHC rats showed a decrease in body weight gain (F_2,97_ = 20.09; p<0.001; p<0.001 vs. both SHC and GHC; Figure 3A) and absolute thymus weight (F_2,97_ = 3.44; p = 0.036; p = 0.050 vs. SHC; p = 0.016 vs. GHC; Figure 3B), whereas absolute adrenal weight (F_2,97_ = 2.09; p = 0.129; Figure 3C) and basal plasma corticosterone (F_2,97_ = 0.28; p = 0.756; Figure 3D) in trunk blood were not different between the groups. Relative thymus (SHC: 1.11±0.06; GHC: 1.12±0.04; CSC: 1.03±0.04; F_2,97_ = 1.43; p = 0.24) and adrenal (SHC: 0.15±0.01; GHC: 0.14±0.00; CSC: 0.15±0.00; F_2,97_ = 0.53; p = 0.590) weights remained unchanged after CSC.

### Experiment 2

#### CSC effects on ACTH and corticosterone responses to an acute heterotypic stressor (open arm)

**Figure 4:**
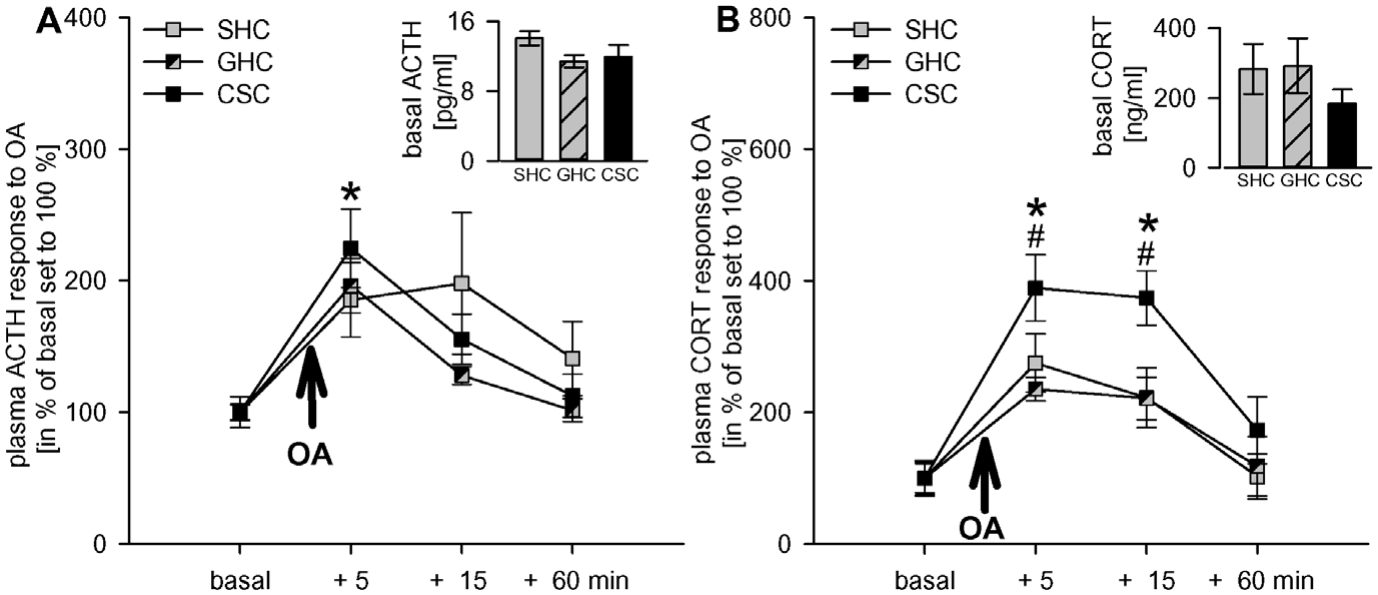
Figure 4. Effects of CSC on plasma ACTH and corticosterone responses to an acute heterotypic stressor. On day 20 after 19 days of CSC exposure, CSC (n = 11), together with single-housed (SHC; n = 11) and group-housed (GHC; n = 10–12) control rats were chronically catheterized. On day 25, i.e. 5 days after recovery from jugular vein catheter surgery, blood was sampled under basal conditions (2 basal 200-µl samples were taken 30 min apart and averaged), and 5, 15 and 60 min after open arm exposure (5 min). Whereas basal plasma ACTH (insert A) and corticosterone (insert B) were not different between the groups, CSC rats showed a more pronounced plasma corticosterone (B) response 5 and 15 min following termination of open arm exposure compared with both SHC and GHC rats, despite comparable ACTH (A) responses between the different groups at all time points measured. Data represent means ± S.E.M.; # p<0.05 versus SHC and GHC, * p<0.05 versus respective basal values.

Open arm exposure (5 min) altered plasma ACTH (factor time: F_3,87_ = 17.11; p<0.001; Figure 4A) and corticosterone (F_3,93_ = 33.90; p<0.001; Figure 4B) levels and increased ACTH levels at 5 min (SHC: p = 0.001; GHC, CSC: p<0.001 versus basal). Similarly, corticosterone levels were significantly increased at 5 (SHC, GHC, CSC: p<0.001) and 15 min (SHC: p = 0.003; GHC: p = 0.002, CSC: p<0.001 versus basal).

CSC prior to open arm exposure did not affect ACTH (factor treatment: F_2,29_ = 0.48; p = 0.622; Figure 4A), but increased corticosterone responses (factor treatment: F_2,31_ = 3.37; p = 0.047; Figure 4B) to open arm exposure. *Post hoc* comparisons revealed that CSC rats had higher plasma corticosterone levels compared with SHC (5 min: p = 0.050; 15 min: p = 0.012) and GHC (5 min: p = 0.009; 15 min: p = 0.010) rats.

In confirmation of results from experiment 1, basal plasma ACTH (F_2,29_ = 1.88; p = 0.171) and corticosterone (F_2,31_ = 0.80; p = 0.457) levels did not differ between groups (see insert, Figures 4A/B, respectively).

### Experiment 3

#### Effects of CSC on colonic damage score, colonic mucus production and IFN-γ secretion from mesenteric lymph node cells

**Figure 5:**
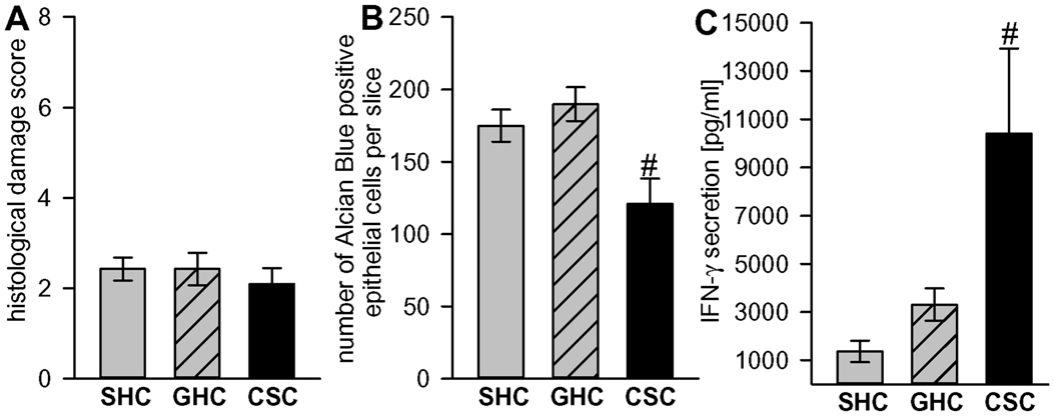
Figure 5. CSC effects on the histological damage score, colonic mucus production, and IFN-γ secretion. Nineteen days of CSC exposure (n = 4–7) did not alter the histological damage score in the colon (A), but decreased the number of Alcian Blue positive epithelial cells in the colon per 20 fields of view (B) and increased IFN-γ secretion from anti-CD3-stimulated mesenteric lymph node cells (C) compared with single-housed controls (SHC: n = 4–7) and group-housed controls (GHC: n = 4–11) rats. Data represent means ± S.E.M.; # p<0.05 versus SHC and GHC.

The histological damage score of the colon was not different between SHC, GHC and CSC rats (F_2,22_ = 0.28; p = 0.759; Figure 5A). However, a decrease in the number of Alcian Blue positive epithelial cells in the colon (F_2,22_ = 7.04; p = 0.004; Figure 5B) of CSC compared with both SHC (p = 0.048) and GHC (p = 0.004) rats provides evidence of mild barrier deficits following CSC exposure. Increased *in vitro* IFN-γ secretion (F_2,9_ = 5.19; p = 0.032; Figure 5C) from anti-CD3-stimulated mesenteric lymph node cells of CSC compared with both SHC (p = 0.014) and GHC (p = 0.04) further indicates intestinal immune activation following CSC exposure.

### Experiment 4

#### Effects of CSC on anxiety- and depression-related behaviours, social preference, and inter-male aggression (Table 1)

The percentage of time spent on the open arms of the elevated plus-maze (F_2,20_ = 2.34; p = 0.122) and in the light compartment of the light-dark box (F_2,21_ = 1.64; p = 0.218), respectively, was not different between SHC, GHC and CSC rats indicating that CSC exposure did not affect general non-social anxiety in rats. With respect to locomotor activity, GHC rats showed an increased number of entries into the closed arms during elevated plus-maze testing (F_2,20_ = 4.91; p = 0.018; p = 0.016 vs SHC), but the number of line crossings/minute in the dark compartment of the light-dark box of did not differ between groups (F_2,21_ = 0.44; p = 0.648).

**Table 1:** Effects of 19 days of CSC on non-social and social anxiety-, depressive-like and aggressive behaviour.

Test	Day	Readout	SHC	GHC	CSC
EPM	20	- time open arm (%)- closed arm entries (n)	14.40±5.85.38±1.31(n = 8)	25.44±4.8811.29±1.27*(n = 7)	29.92±4.998.50±1.36(n = 8)
LDB	21	- time light box (%)- entries dark box/min (n)	13.54±3.2213.73±1.30(n = 8)	16.50±4.87 14.63±1.71(n = 8)	22.84±2.5412.57±1.62(n = 8)
SPAT	23	- social preference (%)	165.41±14.07(n = 8)	154.85±22.93(n = 8)	182.41±26.25(n = 8)
FST	24	- floating events (n)- struggling events(n)- swimming events (n)	69.25±3.1539.50±3.2811.25±2.01(n = 8)	71.00±1.1836.25±3.1212.75±3.06(n = 8)	72.75±4.7839.63±3.297.63±2.38(n = 8)
RI test	30	- time aggression (%)- attack latency (s)- number of attacks (n)	22.19±7.06424.58±64.012.28±0.92(n = 8)	18.93±5.72461.17±69.165.0±2.38(n = 8)	20.76±5.48473.09±47.661.0±0.44(n = 7)

CSC did not alter non-social anxiety on the elevated plus-maze (EPM; day 20) and in the light-dark box (LDB; day 21). Social anxiety as evaluated in the social preference/avoidance test (SPAT; day 23), stress-coping behaviour as assessed in the forced swim test (FST; day 24), and inter-male aggression as measured during the resident-intruder test (RI test; day 30) were not affected by CSC. Group size is presented in parentheses. Data represent mean ± S.E.M.

* p<0.05 versus SHC.

Furthermore, the number of floating events (passive stress-coping behaviour) during the forced swim test, indicative of depression-like behaviour (F_2,21_ = 0.27; p = 0.767), as well as the number of struggling (F_2,21_ = 0.35; p = 0.708) and swimming events (F_2,21_ = 1.09; p = 0.35), indicative of active stress-coping behaviour, were not different between SHC, GHC and CSC rats.

In addition, CSC exposure did not affect social preference behaviour (F_2,21_ = 2:79; p = 0.085), and did not induce signs of social avoidance.

Finally, no differences were found in the percentage time CSC rats (here used as residents during the resident intruder test on day 10 after CSC) spent with aggressive behaviour (F_2,20_ = 0.02; p = 0.977), in the attack latency (F_2,20_ = 0.06; p = 0.946), and in the number of attacks (F_2,20_ = 1.82; p = 0.189) during the RI test between SHC, GHC and CSC rats, indicating no CSC effect on inter-male aggression.

## Discussion

Our study shows that chronic subordinate colony housing (CSC) is an efficient chronic psychosocial stress paradigm also in male Wistar rats. In addition to a decrease in body and absolute thymus weights observed after 19 days of CSC, there are several other physiological, immunological and behavioural parameters which were found to be comparable between chronically stressed rats and mice [12], [15]. Thus, CSC rats showed increased IFN-γ secretion from isolated and anti-CD3-stimulated mesenteric lymph node cells, a decreased number of mucus producing epithelial cells in the colon, and unaffected basal plasma corticosterone levels, whereas, most importantly, plasma corticosterone response to subsequent acute open arm exposure was found to be exaggerated. Further in line with our mice data, CSC rats neither showed a reduction in social preference nor increased passive stress coping behaviour in the forced swim test, but, and this is in contrast to CSC mice, stressed rats did not show an increase in general anxiety-related behaviour.

Extending the finding of a more pronounced corticosterone response to a heterotypic stressor to another species, namely rats, in the present study substantially strengthens our hypothesis drawn from recent mouse data [18]. Thus, CSC-induced changes at the level of the adrenal gland, i.e. increased *in vivo* ACTH responsiveness during acute heterotypic stressor exposure, but no significant changes in basal plasma corticosterone levels may represent a beneficial adaptation to, rather than a maladaptive consequence of chronic psychosocial stressor exposure. This allows a chronically stressed organism to adequately respond to a novel threat, while preventing prolonged exposure to high basal levels of deleterious glucocorticoids. Thus, the present study provides support for the general value of the CSC paradigm to reveal physiological or behavioural adaptations as a consequence of chronic psychosocial stress not only in mice, but also in rats.

### Choice of Adequate Control Group

For meaningful data interpretation, it is essential to choose an adequate control group. As this issue is still controversial for rats, we decided to consistently employ both SHC and GHC rats as adequate controls. This is in contrast to studies performed in male mice, where we [14] and others [39], [40] convincingly showed that single housing is an adequate and stress-free housing condition lacking any stress-relevant behavioural, physiological or immunological symptoms. However, there are several studies demonstrating that single housing of rats *per se* is stressful and may result in anxiety- and depressive-like phenotypes [41], [42], as well as in major deficits in sexual behaviour [43]. One possible explanation might be that the consequences of housing conditions are strain- [44] or sex- specific [45], [46]. Of considerable importance seems to further be the duration of social isolation, which varies in the above mentioned studies from 8 to 15 days [42], [45] up to 10 or even 13 weeks [41], [43], [46]. Given that SHC and GHC rats in the current study did not differ in any physiological, neuroendocrine or immunological parameters investigated, we are convinced that both control groups can still be employed, with consideration of the above mentioned factors.

### Confirmation of Chronic Stress Consequences

For validation of a chronic stress model, for example in a novel species, several key parameters need to be affected. For example, a decrease in body weight gain, as observed in the subordinate male CSC compared with both SHC and GHC rats, is a relevant physiological marker of chronic psychosocial stress [12], [47]–[49].

Another important indicator for CSC depicting a relevant chronic psychosocial stress model for male rats [50], [51] is the development of a pronounced thymus atrophy which we could find in CSC compared with both SHC and GHC rats. Previous studies employing 24-h social defeat stress suggested that thymus atrophy is mediated by a decrease in the number of thymocytes, specifically the CD4^+^ and CD8^+^ subpopulation [11], [50] and, thus, reduces the functional capacity of T cells. Given that in this respect glucocorticoids have been shown to play an important role [52], we hypothesize that, like in CSC mice [12], increased plasma corticosterone levels during the initial phase of CSC exposure may initiate thymus atrophy. Moreover, the medullary part of the thymus exhibits a high density of ß-adrenergic receptors [53] involved in cAMP-mediated thymocyte apoptosis [54] and loss of thymus mass [50]. Although further studies need to investigate this in detail, it is further likely that the sympathetic-adrenomedullary system is chronically activated [55], [56] in CSC rats. Therefore, glucocorticoid-initiated thymus involution could be maintained during CSC exposure by an increased drive of the sympathetic nervous system.

Although adrenal hypertrophy is generally referred to as an accepted indicator of chronic stress in rats and mice [5], [57], and has recently been shown in CSC mice [18], there are studies showing that especially in rats, an increase in adrenal mass is not a reliable biomarker for chronic stress. For instance, 19 to 28 days of subordination during colony housing in male Wistar rats did not result in adrenal hypertrophy, despite a decreased body weight gain and thymic involution [51]. Furthermore, exposing rats to the visible burrow system as an accepted animal model of chronic psychosocial stress does not reliably result in an increase of adrenal mass [10]. Support comes also from our own recent data, showing that repeated restraint and overcrowding stress increases adrenal mass in lactating female rats, but not in virgins [58]. Thus, the lack of a CSC effect on absolute rat adrenal mass as seen in the present study is in contrast to what is repeatedly described for CSC mice, but is in line with other rat studies showing no effect of chronic stress on adrenal weight. Further studies need to be carried out; perhaps even in different rat strains, to properly dissect the underlying adrenal mechanisms during CSC exposure in the rat.

In contrast to the species-dependent effects of CSC exposure on absolute adrenal mass in mice and rats, CSC did not affect basal, but did affect acute stress-induced HPA axis function in both species. In detail, comparable to CSC mice [12], [18], CSC rats showed unaffected basal morning plasma corticosterone levels, but an exaggerated plasma corticosterone response to an acute heterotypic emotional stressor, relevant for rats [59], i.e. 5-min exposure to the open arm of an elevated plus-maze (compared with both SHC and GHC rats). Given that plasma ACTH response to open arm exposure was comparable between SHC, GHC, and CSC rats, this suggests either an increased *in vivo* ACTH responsiveness of the CSC rat adrenal cortex during acute heterotypic stressor exposure or glucocorticoid secretion to be driven by an additional corticosterone secretagogue different from ACTH. Again, this is strongly in line with our mouse CSC data, showing an increased corticosterone, but unchanged ACTH secretion in response to acute elevated platform exposure following CSC [18]. However, in contrast to CSC mice, absolute adrenal mass is not increased in CSC rats. This suggests development of morphological/histological/cellular adrenal changes different from just an increase in absolute adrenal weight, enabling rat CSC adrenals to show an exaggerated response to acute heterotypic stressors. Given that basal plasma corticosterone levels are not increased in CSC rats, adrenal ACTH responsiveness and/or release of the above mentioned corticosterone secretagogue seems to be enhanced specifically during acute stress. This allows a chronically stressed organism to adequately respond to a novel threat, but protects it from being exposed to deleterious consequences of basally elevated plasma corticosterone levels. However, detailed investigation is required to confirm the *in vitro* reactivity of the rat adrenals to ACTH.

Increased secretion of the pro-inflammatory cytokine IFN-γ from isolated mesenteric lymph node cells and decreased colonic sulfomucins have turned out to be reliable and sensitive indicators of mild colonic inflammation and breakdown of epithelial barrier function, induced by CSC exposure in mice [12], [16], [48]. Similarly, the finding of increased IFN-γ secretion from isolated mesenteric lymph node cells together with the decreased number of colonic sulfomucin positive epithelial crypt cells in CSC compared with SHC and GHC rats in the present study suggest that CSC, in a species-independent manner, causes colonic barrier defects coupled with intestinal immune activation. Additional studies are needed to characterize CSC-induced cellular alterations in the colon in more detail, by investigating, for instance, mast cell activation/infiltration and mechanisms of intestinal barrier dys-functions as performed in comparable studies following chronic crowding stress in rats [60], [61]. However, the lack of detectable histological damage in CSC rats, which is in contrast to our findings in CSC mice [12], may either represent a species-specific protection of epithelial barrier function, or higher stress resilience in male Wistar rats compared with male C57Bl6 mice.

The latter is further supported by the fact that CSC rats - in contrast to CSC mice [12]–[15], [18], [62], [63] - do not develop increased anxiety-related behaviour, which has been monitored right at the end of (elevated plus-maze, day 20 of CSC) or one day after termination of CSC exposure (light-dark box, day 21). We have recently shown that elevated levels of anxiety-related behaviour can appear as late as 9 days following termination of chronic psychosocial stress in mice [15]. Therefore, the possibility exists that alterations in anxiety-related behaviour develop at a later time point in CSC rats. Support for this option comes from McCormick and colleagues [64] showing increased anxiety-related behaviour on the elevated plus-maze in male rats 25 days after termination of a 2-week social stress procedure. Although the lack of behavioural alterations following chronic stress exposure is rather untypical in rodent studies, it offers the unique possibility to dissect the mechanisms underlying stress-induced somatic pathologies without conflicting affective alterations. However, future studies are needed to assess CSC effects on anxiety-related behaviour also at later time points.

Our finding of unchanged social preference behaviour in the social preference/avoidance test and stress-coping behaviour in the forced swim test following CSC in rats are well in line with our recent mice studies [15]. Moreover, CSC rats displayed adequate aggressive behaviour, reflected by unchanged attack latencies and number of attacks during the resident intruder test comparable to those seen in both control groups. Whether the unknown con-specific is confined in a wire mesh cage (as in the social preference/avoidance test) or interacts as intruder actively with the experimental animal (as in the resident intruder test), CSC exposure does not seem to influence both the passive and active social interaction behaviour of these animals.

In summary, our results further confirm and extend the validity of the CSC paradigm as a relevant chronic psychosocial stressor also in male Wistar rats. The present study reveals that CSC rats (i) show adaptive, rather than mal-adaptive HPA axis changes at the level of the adrenal gland, (ii) develop mild colonic barrier defects and intestinal immune activation and (iii), show no anxiety-like behaviour, no increase in passive stress-coping, no increase in aggression, and do not lose their social preference.
